# The metacoupled Arctic: Human–nature interactions across local to global scales as drivers of sustainability

**DOI:** 10.1007/s13280-022-01729-9

**Published:** 2022-03-30

**Authors:** Kelly Kapsar, Veronica F. Frans, Lawson W. Brigham, Jianguo Liu

**Affiliations:** 1 Department of Fisheries & Wildlife, Center for Systems Integration and Sustainability, Michigan State University, 115 Manly Miles Building, 1405 S. Harrison Rd., East Lansing, MI 48823 USA; 2 International Arctic Research Center, University of Alaska Fairbanks, PO Box 757340, Fairbanks, AK 99775-7340 USA

**Keywords:** Climate change, Complexity, Globalization, Human–environment systems, Social–ecological systems, Telecoupling

## Abstract

The Arctic is an epicenter of complex environmental and socioeconomic change. Strengthened connections between Arctic and non-Arctic systems could threaten or enhance Arctic sustainability, but studies of external influences on the Arctic are scattered and fragmented in academic literature. Here, we review and synthesize how external influences have been analyzed in Arctic-coupled human and natural systems (CHANS) literature. Results show that the Arctic is affected by numerous external influences nearby and faraway, including global markets, climate change, governance, military security, and tourism. However, apart from climate change, these connections are infrequently the focus of Arctic CHANS analyses. We demonstrate how Arctic CHANS research could be enhanced and research gaps could be filled using the holistic framework of metacoupling (human–nature interactions within as well as between adjacent and distant systems). Our perspectives provide new approaches to enhance the sustainability of Arctic systems in an interconnected world.

**Supplementary Information:**

The online version contains supplementary material available at 10.1007/s13280-022-01729-9.

## Introduction

The Arctic is a diverse region with many complex environmental and socioeconomic systems. Researchers attempting to understand these systems have frequently applied a coupled human and natural systems (CHANS) framework (Liu et al. 2007; Alberti et al. 2011). Also known as social–ecological or human–environment systems research, CHANS approaches examine not only environmental (e.g., ecosystems, hydrological systems) or human (e.g., governments, social networks) systems, but also the human–nature interactions that bind them together.

Arctic CHANS research has been at the forefront of several advances in CHANS approaches, including increased integration of human and natural systems as well as the incorporation of traditional and local knowledge (Petrov et al. 2016). Examples of Arctic CHANS studies include resilience assessments of the impacts of natural resource development on reindeer herding practices in Russia (Forbes et al. 2009), participatory mapping of environmental change by Indigenous communities in northern Canada (Gill et al. 2014), and an analysis of the impacts of fisheries privatization in Iceland (Kokorsch and Benediktsson 2018), among others.

However, Arctic CHANS do not operate in isolation. They are frequently impacted by actions occurring in and with adjacent and/or distant systems. For example, one of the main linkages between the Arctic and the rest of the world is through the influence of greenhouse gas emissions on the Arctic climate. Climate change, a phenomenon primarily fueled by greenhouse gas emissions from lower latitudes, is causing the Arctic to warm at more than twice the global average rate (Overland et al. 2019). These rising temperatures have cascading effects on Arctic ecosystems and their human residents through mechanisms such as thawing permafrost (Shi et al. 2019), reducing sea ice extent (Parkinson 2014), and altering the migration timing and patterns of wildlife, which subsequently changes the seasonality and location of subsistence harvests (Kovacs et al. 2011).

Aside from climate change, numerous other external influences, such as natural resource development and global markets, have complex effects on the sustainability of Arctic CHANS. For example, the development of oil and natural gas extraction can have positive impacts on the job opportunities and economic wellbeing of isolated communities, as was the case in Hammerfest, Norway after offshore oil development began (Loe and Kelman 2016). However, oil exploration can also negatively impact flora and fauna, such as those in Alaska, where regions proposed for offshore drilling substantially overlapped with cetacean habitats (Reeves et al. 2014).

While connections between the Arctic and lower latitudes are not new, their strength and frequency have dramatically increased in recent decades. These growing connections indicate that the sustainability of Arctic regions could be increasingly influenced by distant actors, foreign policies, and global markets (National Research Council 2015; Callaghan and Johansson 2021). This pattern has sparked concern among Arctic residents and policymakers alike and has resulted in calls for an increased understanding of the complex, interactive effects of multiple external influences operating within or affecting Arctic systems (Members of the World Economic Forum Global Agenda Council on the Arctic 2014; Larsen and Fondahl 2015). To better understand the complex nature of these external connections there is a need for comprehensive conceptual frameworks that incorporate the interactions between multiple CHANS.

In recent years, the conceptual framework of metacoupling has emerged as one such tool. The framework of metacoupling organizes CHANS into five component parts (systems, agents, flows, causes, and effects) for the purpose of categorizing and better understanding system sustainability (Table 1; Liu 2017; Liu et al. 2021). Metacoupled CHANS include three types of couplings based on the number of systems and their relationships to each other: intracouplings, pericouplings, and telecouplings. Intracouplings are socioeconomic and environmental interactions within a single system; pericouplings occur when socioeconomic and environmental interactions occur between adjacent systems; and telecouplings occur when these interactions form between distant systems. The term metacoupling is an umbrella concept that encompasses all three types of couplings (intra-, peri-, and telecoupling).

**Table 1:** Description of the five components of the metacoupling framework with common examples and relevant literature focusing on each component

Components of the metacoupling framework	Definition of metacoupling component	Examples	Relevant literature
Sending systems	Systems in which a given flow originates	• Community/Village	• Friis and Nielsen (2017)
		• Region	• Liu et al. (2015)
		• Biodiversity hotspot	• Andriamihaja et al. (2019)
		• Country	• Herzberger et al. (2019)
Telecoupled receiving systems	Distant systems in which a given flow terminates	• Importing countries	• Sun et al. (2018)
		• Tourist destinations	• Yao et al. (2020)
Pericoupled receiving systems	Adjacent systems in which a given flow terminates	• Seasonal migration destinations	• Hulina et al. (2017)
		• Intermediate processors	• Herzberger et al. (2019)
Spillover systems	Systems that affect or are affected by the flow or its transportation from sending to receiving systems	• Coastal areas	• Liu et al. (2018)
		• Downstream or neighboring ecosystems/communities	• Zhao et al. (2020)
Flows	Movement of materials, energy, or information	• Animal migration	• López-hoffman et al. (2017)
		• Tourism	• Chung et al. (2020a)
		• Trade	• Xiong et al. (2018)
		• Technology transfer	• Tonini and Liu (2017)
		• Investment	• Yang et al. (2016)
		• Human migration	• Zimmerer et al. (2018)
		• Knowledge transfer	• Carlson et al. (2017)
		• Species dispersal	• LaRue et al. (2021)
		• Water transfer	• Deines et al. (2016)
		• Waste transfer	• Liu et al. (2014)
Agents	Individual actors or institutions involved in the development, maintenance, or termination of a metacoupled flow	• Community members	• Liu and Agusdinata (2021)
		• Policy makers	• Yang et al. (2018)
		• Regulators	• Kalt et al. (2021)
		• NGO representatives	• Andriamihaja et al. (2019)
		• Industry representatives	• Marola et al. (2020)
Causes	Environmental, socioeconomic, political, or technological drivers that work to initiate a flow within or between systems. Can occur in sending, receiving, or spillover systems	• Demand for resources	• Carlson et al. (2017)
		• Natural disaster	• Zhang et al. (2018)
		• Policy implementation	• Herzberger et al. (2019)
Effects	Environmental, socioeconomic, political, or technological impacts of a metacoupling process. Can occur in sending, receiving, or spillover systems	• Land use/Land cover change	• da Silva et al. (2021)
		• Improved/diminished wellbeing	• Llopis et al. (2020)
		• Biodiversity change	• Kuemmerle et al. (2019)

This framework builds upon existing conceptual frameworks, such as Ostrom’s approach to sustainable social–ecological systems, which are examples of CHANS (Liu et al. 2007), by explicitly incorporating the reciprocal influences of external connections to focal system(s) (Ostrom 2007). These external connections to Arctic CHANS are a critical element of CHANS analyses, as they pose a potential challenge for the sustainable governance of common pool resources by violating the first design principle of defining a clear and closed set of resource users (Ostrom 1990). Additionally, when resources are primarily extracted for use in systems that are considered “exogenous” to a focal system (e.g., oil and gas exports from the Arctic), it is unreasonable to assume that the sustainability of a focal system can be achieved in isolation from those to which it is tightly linked through socioeconomic and environmental flows.

To understand the complex interactions between Arctic CHANS and other regions and their impacts on Arctic sustainability, it is necessary to first take stock of studies that have incorporated external influences in existing analyses. To this end, we conducted a literature review of studies analyzing Arctic systems as CHANS. After identifying the state of Arctic CHANS analyses from our literature review, we highlight the potential for using the framework of metacoupling (human–nature interactions within as well as between adjacent and distant systems; Liu 2017) as a method for integrating, advancing, and communicating CHANS research in the Arctic (Liu 2017; Liu et al. 2021). To demonstrate its application to Arctic systems, we provide examples of metacouplings in the Arctic CHANS analyses from our literature review and from other Arctic research. We purposefully chose to illustrate numerous examples from many areas of the Arctic CHANS literature to demonstrate the broad applicability of the metacoupling framework and to lay the groundwork for future analyses. We then discuss ways in which the metacoupling framework can be used to identify the cumulative and interactive effects of multiple external influences, as well as to identify gaps in the literature and new potential areas of knowledge synthesis.

## Literature review of Arctic CHANS research

### Methods of literature review

To conduct a systematic review of the Arctic CHANS literature regarding external influences, we ran a Web of Science topic search using the following sets of terms: (1) “social–ecological” AND “Arctic”; (2) “socioecological” AND “Arctic”; (3) “coupled human and natural” AND “Arctic”; (4) “human-rangifer” AND “Arctic”; and (5) “human–environment” AND “Arctic”. Topic searches identified all articles containing the search terms in the title, keywords, or abstract. We chose these search terms to represent the varied terms used to refer to the study of CHANS.

We screened the abstracts and methods sections of articles identified in the Web of Science search and selected relevant articles based on four criteria. First, we excluded papers conducting studies outside the boundaries of the Arctic as defined by the Arctic Monitoring and Assessment Program (Arctic Monitoring and Assessment Programme (AMAP) 1998). Second, we excluded papers that did not conduct qualitative or quantitative analyses (e.g., conceptual papers, reviews, or papers without explicitly described methodologies), as we were primarily interested in the degree to which external influences were incorporated in the data collection process. We identified analytical papers as those that specifically described the methods used to develop or aggregate the information presented. We included papers presenting a case study based on a conceptual framework or meta-analysis if data collection methods were present. We also excluded book chapters and gray literature. Third, we excluded papers that did not use CHANS language (see above search terms) in the context of discussing the study system. This approach alleviated the need to make arbitrary decisions about the study’s qualification as CHANS research using the study authors’ self-designated definition of the system as a CHANS. Lastly, we excluded papers that did not use a CHANS approach (e.g., ecological analyses that acknowledge the system is a CHANS). To ensure that all relevant articles were identified, even if they were not in the search results, we conducted forward and backward reference checks using a snowballing method for all studies retained after initial screening (Wohlin 2014). All papers published through June of 2020 that fit the screening criteria were included in the analysis.

From each paper, we collected the number of countries studied, the name(s) of the Arctic country or countries studied, the type of research, the geographic scale(s) of the research, and the degree of community involvement in the research (Table S1). We also used the research questions, study area description, methods, and abstract to determine whether an analysis of external influences was a primary focus of the paper, and used inductive coding to categorize these external influences into groups (Thomas 2006).

### External influences identified in the analyses of Arctic CHANS

**Fig. 1:**
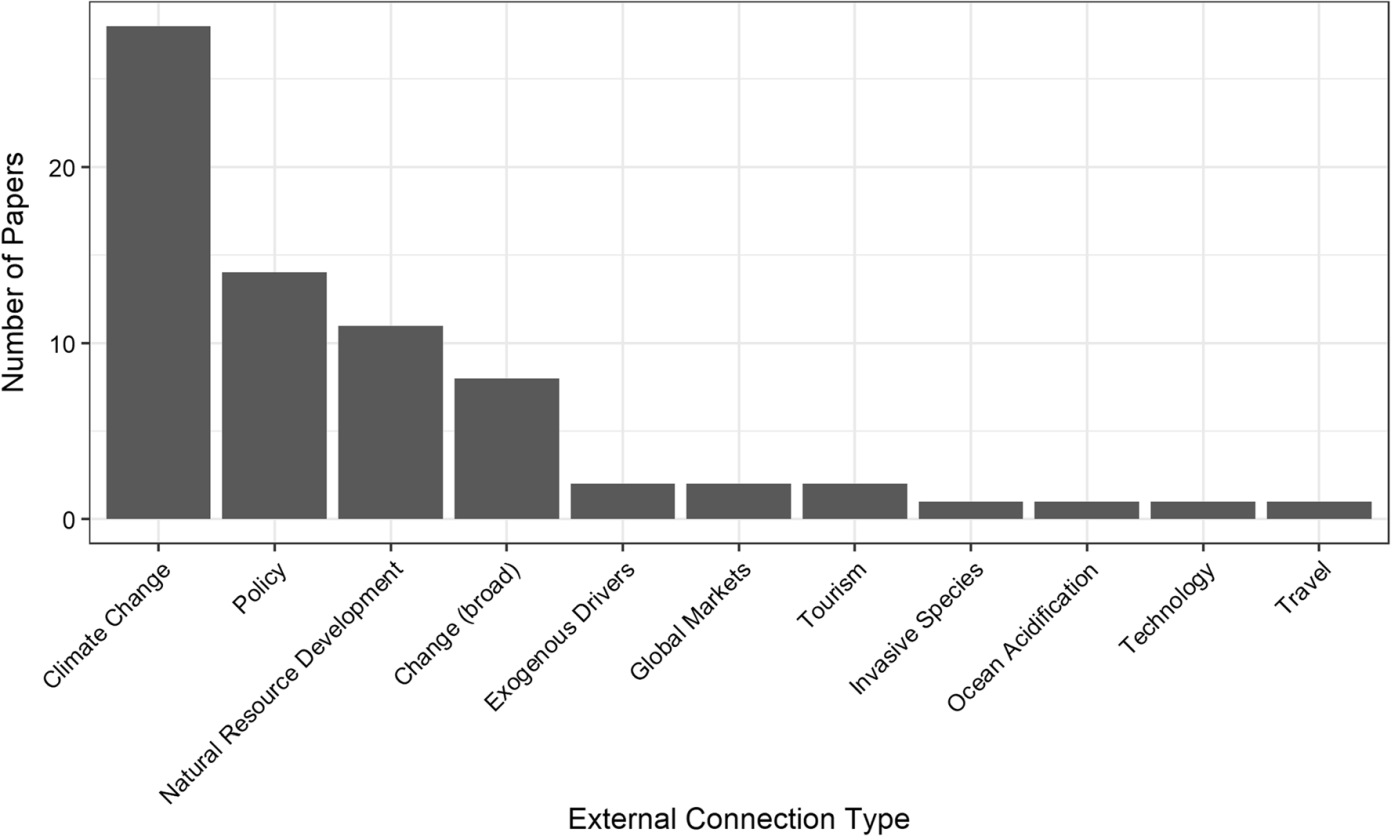
Most common external influences in Arctic CHANS analyses. Note that papers can have more than one external influence

We identified a total of 103 studies conducting an analysis of the Arctic as a CHANS (Table S2). External influences were a focus of analysis in 61% of these Arctic CHANS studies. Climate change was the most commonly studied external influence (Fig. 1). Other common external influences included policy (11% of studies) and natural resource development (8%). Eight percent of studies focused on “change” in a broadly defined way that included external influences (e.g., broadly speaking about globalization). Global markets, invasive species, trade, technology transfer, and tourism were each the focus of research in less than 3% of studies. While a few studies examined Arctic CHANS as one of multiple case studies, no study explicitly examined the connection between an Arctic CHANS and one or more non-Arctic CHANS.

In our study sample, academic research on external influences appeared to be heavily focused on climate change. Climate change was the sole external influence studied in 22 of the 64 studies that analyzed at least one external influence. While not the primary focus of analysis, other types of external influences, such as international trade, natural resource development, governance, and tourism, were often discussed by Arctic residents in interviews (Moerlein and Carothers 2012; Ford et al. 2013). For example, when Moerlein and Carothers (2012) asked Inupiaq elders in northwestern Alaska about the environmental impacts of climate change, they found that residents holistically incorporated both social and environmental change into their responses. This observation contrasts with traditional academic approaches that treat social and environmental problems as separate. In their conclusion, Moerlein and Carothers state that “these communities face a total environment of change, whereby environmental changes and broader socioeconomic challenges are jointly shifting and remaking human–environment relationships” (Moerlein and Carothers 2012). Results such as these demonstrate the need for more integrative frameworks that can be applied to examine the socio-environmental interactions and feedback effects of multiple external influences on Arctic CHANS.

Similar to the types of external influences, the scales of analysis and geographic distribution of Arctic CHANS research in our sample were also skewed. Most Arctic CHANS analyses took place at the regional (within-country) extent (54%). These studies typically presented the aggregated results and/or a comparison of results of data collected from several focal communities. Single community studies were the second most frequent scale of analysis (22%), followed by studies with multiple scales of analysis (14%), international studies (9%), and national scale studies (1%). The USA was the most common study location for Arctic CHANS analyses (50 studies), followed by Canada (25), Norway (22), and Russia (17). Finland, Sweden, Iceland, and Greenland (Denmark) collectively had fewer than 7 studies.

Qualitative methods were the most common form of analysis, comprising 45% of studies, followed by mixed qualitative and quantitative (35%), and quantitative (20%) studies. Over 75% of studies involved local communities in some manner. The most common form of involvement for local communities was as participants in data collection, with co-design and/or co-production of knowledge described in only 23% of studies.

External influences on the Arctic have been analyzed in numerous disciplinary and even multidisciplinary combinations (e.g., climatology (Overland and Wang 2018), climatology and economics (Petrick et al. 2017), climatology, economics, and fisheries science (Eide 2008)). However, despite increasing calls from researchers and policymakers for more interdisciplinary research on Arctic systems (Arctic Council 2016; Petrov et al. 2016; Anderson et al. 2018), our review demonstrates that external influences, particularly those other than climate change, are infrequently the focus of analysis in the Arctic CHANS literature. Less than half of the papers in our review analyzed an external influence that was not climate change.

This finding indicates a need for more analyses of the interconnections between Arctic and non-Arctic systems and their implications for the sustainability of Arctic CHANS. To this end, we present the conceptual framework of metacoupling and describe how it can be used to synthesize knowledge on connections between multiple CHANS and their effects on sustainability in the Arctic.

## Application of the metacoupling framework

To promote more studies on the interactions between Arctic and non-Arctic systems and to address the lack of integration of external influences in the Arctic CHANS literature, we suggest the application of the metacoupling framework. This framework builds upon CHANS research, as well as scholarship related to the distant connections between CHANS, known as telecouplings (Liu 2017; Kapsar et al. 2019). Stemming from the field of geography and other fields such as ecology and socioeconomics, the metacoupling framework allows researchers to integrate disciplinary research into interdisciplinary understandings of complex systems. The framework is general and can be applied to any CHANS although the specifics (e.g., agents, flows, effects) may differ. For example, the framework has been applied to global marine fishing (Carlson et al. 2020), freshwater ecosystem services to global cities (Chung et al. 2021), and impacts of international trade on global sustainable development (Xu et al. 2020a). In different systems, we would expect that the unique socioeconomic and environmental contexts would lead to different system structures and sustainability outcomes under the metacoupling framework. However, comparative studies between metacoupled systems could help to identify similarities and differences as well as important structures that facilitate or hinder sustainability objectives.

**Fig. 2:**
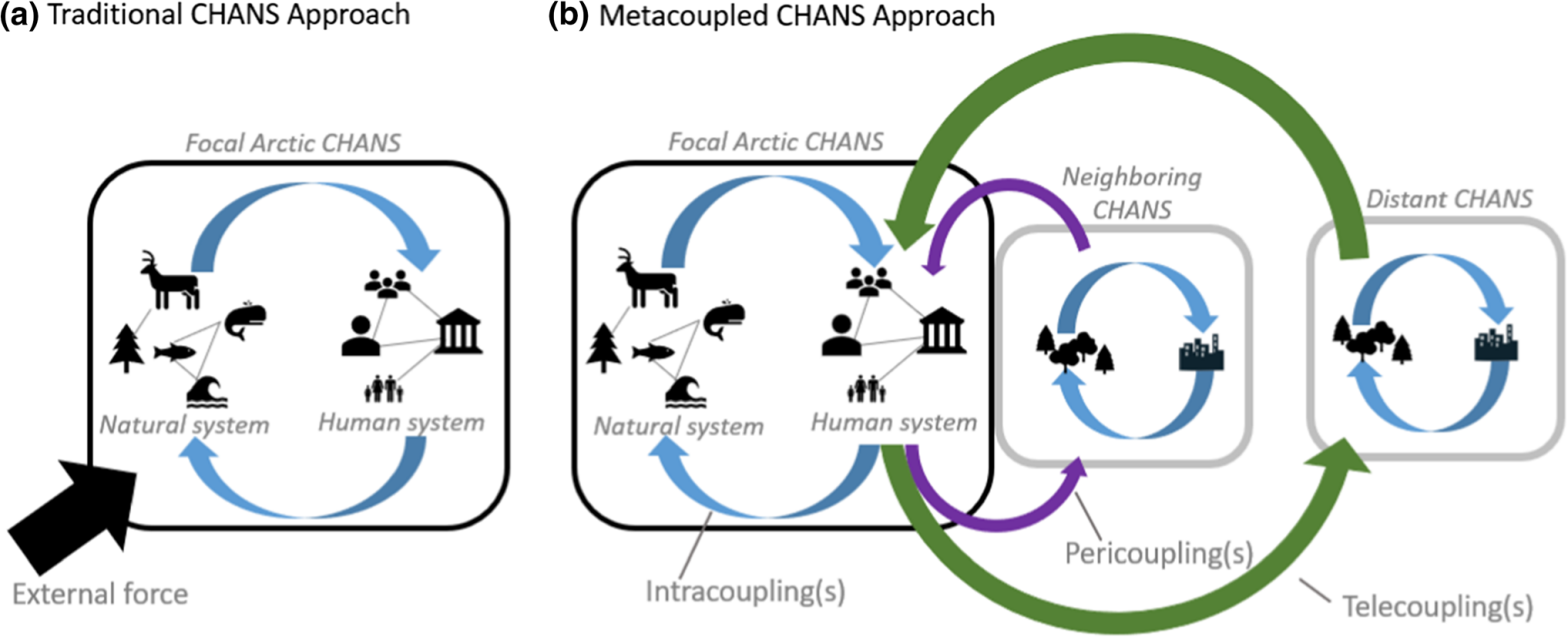
Conceptual diagram demonstrating the differences between a traditional CHANS and a metacoupled CHANS approach to analyzing the effects of external forces on Arctic systems. Focal systems are outlined in black while non-focal systems are outlined in grey. Blue arrows represent intracoupled flows of materials, information, people, and/or energy. Purple arrows represent pericoupled flows (between neighboring CHANS). Green arrows represent telecoupled flows between distant CHANS. In addition to external influences on Arctic systems, the metacoupled approach also considers the impacts of the Arctic on other systems (e.g., feedback, provision of Arctic resources to lower latitudes, cold spells and heavy precipitation to lower latitudes)

As a conceptual construct, the metacoupling framework serves to guide researchers in situating their research within a broader context by taking into account transboundary socioeconomic and environmental interactions in a systematic manner (Fig. 2). Similar to the way the umbrella concept of “ecosystem services” has integrated disciplinary knowledge of the benefits of the natural environment for humanity, the metacoupling framework can be applied to synthesize knowledge of diverse connections between CHANS and their impacts on social–ecological sustainability.

The metacoupling framework provides several conceptual advances that can build upon previous Arctic CHANS scholarship, such as the explicit incorporation of feedback effects. Previous research on the role of external influences on CHANS has examined their role in shaping system sustainability in a unidirectional way. For example, when analyzing the influence of exogenous drivers on Indigenous subsistence communities in the western Arctic, Fauchald et al. (2017) distinguish between exogenous drivers that act directly on a natural resource (e.g., commercial fishing) and those that act on resource users (e.g., technology access). The metacoupling framework builds upon this foundational knowledge of exogenous drivers through the incorporation of feedback effects, whereby actors influence the driver itself or the system from which the driver originates. Feedback effects are commonly studied in complex adaptive systems like CHANS (Levin et al. 2012), and are a key aspect of metacoupled systems (Hull et al. 2015; Yang et al. 2018).

Another conceptual advancement of the metacoupling framework is the explicit incorporation of external systems and cross-scale interactions. In our review of Arctic CHANS studies, many analyses acknowledged the role of external forces (Fig. 2a). For example, ten studies had analytical approaches focused on broadly defined “change” or “exogenous drivers”. These studies often fail to account for the scale of operation of that driver (e.g., global, regional, local), the distance between exogenous drivers and the focal system, and/or the relative orientation between the interconnected systems (e.g., neighboring, distant). Furthermore, the metacoupling framework facilitates studies regarding not only impacts of adjacent and distant systems (e.g., lower-latitude regions) on the focal system (e.g., Arctic), but also impacts of the focal system on other systems nearby and far away.

Scale and geographic proximity of systems may play a more significant role in certain metacoupling types than others. For example, information and governance decisions can be transmitted across long distances in very short time spans over the internet. Thus, the distance between the sending and receiving systems (and thus the definition of peri- vs. telecoupling) may not be as critical under some circumstances. However, in the context of the marine transportation of oil and gas from northern Russia to Asian ports, the voyage distance**s** between sending and receiving systems is a very relevant factor that influence the rate of transportation of the flow as well as the spillover environmental effects of marine shipping.

Timescale is also an important part of metacoupling processes. Metacoupling processes are dynamic over time, such as with anthropogenic climate change, and different metacoupling processes take place across dramatically different timescales. For example, crop domestication takes place over years to centuries, while financial transfers can complete over seconds. Additionally, metacoupling processes may exhibit common trajectories of formation, growth, and dissolution over time (Liu 2017), however this is an emerging area of metacoupling research that is in need of further study.

The metacoupling framework explicitly identifies five components of CHANS: *flows, systems, agents, causes* and *effects* (Fig. 2b; Table 1). *Flows* are defined as the movement of materials, information, or energy within or between metacoupled system(s). Flows can be both material (e.g., copper, nickel, oil) or immaterial (e.g., information). For instance, in the case of commercial fishing, the flow would be the movement of fish; in the case of pollution, the flow would be the movement of the pollutant; and in the case of policy implementation, the flow would be the movement of information. Flows are frequently associated with feedback effects that work to strengthen (positive) or weaken (negative) the original flow (e.g., Yang et al. 2018). *Systems* are the CHANS in which the metacoupling processes take place. They can be sending systems if the metacoupled flow originates from them, receiving systems if the metacoupled flow is sent to them, or spillover systems if they are impacted by the metacoupling processes, such as interactions between sending and receiving systems. *Agents* are the entities involved in the transfer of those flows. Agents could be the regulating authorities controlling the flow or the flows themselves (e.g., migratory wildlife). *Causes* are the human and/or natural factor(s) that initiate a metacoupling process, and *effects* are the outcomes of a metacoupling process within all involved systems.

Researchers can compare the components of the framework and interrelationships among them with the human and natural components and their interrelationships within a particular metacoupled system. Comparative analyses can help identify knowledge gaps and generate hypotheses about the relevant links. While there is no single prescribed methodology for identifying metacoupled system components, commonly applied methods from previous studies include literature review, field work, and qualitative research. For example, Friis and Nielsen (2016) used ethnographic field research to examine local communities’ perceptions of telecoupled foreign investments in banana plantations in Laos. In addition, a wide variety of methods have been used to analyze telecoupled flows, causes, and effects, including network modeling and cluster analysis (Chung et al. 2020b), agent-based modeling (Dou et al. 2019), time series analysis (Carlson et al. 2020), life-cycle analysis (Xu et al. 2020b), and remote sensing of land use and land cover change (Leisz et al. 2016).

Not all components of a given metacoupling process may be relevant in all studies. Intracoupling processes may exist in isolation from telecouplings and vice versa. Or, there may not be significant spillover effects related to a given pericoupling process. In this way, the metacoupling framework is not a panacea, but rather a tool that can be applied to unearth potential new aspects of a given system or to examine the effects that changes in one system could have upon other systems.

Below, we describe each metacoupling type in turn, discuss how they relate to existing Arctic CHANS analyses or studies, and provide further examples of existing research reframed under the conceptual framework of metacoupling. In addition, we discuss challenges for Arctic metacoupling research and the value of the metacoupling framework to Arctic sustainability research and policy.

### Arctic intracouplings

Intracouplings are human–nature interactions that occur within a system, such as the subsistence harvest of plants and wildlife inside Arctic CHANS. In our literature review, community and regional-scale studies comprised over three quarters of the studies we analyzed. Moreover, over three quarters of analyzed studies involved the input of local communities in some manner, indicating that intracouplings are a key topic of academic research interest in the Arctic. These findings are expected given the prevalence of pastoralist and subsistence livelihoods in the circumpolar Arctic. In many parts of Alaska, for example, Indigenous communities maintain the traditional subsistence harvest of well over 50% of the foods that they consume (Fall 2016). The subsistence way of life, practiced by Indigenous communities for thousands of years, is interdependent with a healthy ecosystem that can support the large mammals harvested by many communities as key elements of their diet and cultural wellbeing. Additionally, the concept of food security in Arctic Indigenous communities is inextricable from the practice of subsistence (Inuit Circumpolar Council—Alaska 2015). When viewed through the metacoupling framework, subsistence can be considered an intracoupled process or intracoupled human–environment interaction. While subsistence is one of the most straightforward intracouplings in the Arctic, other examples include farming, forestry, and environmental restoration, among others.

### Arctic pericouplings

Pericouplings are human–nature interactions between adjacent-coupled systems. The interconnected nature of the Arctic has resulted in the development of many pericoupled processes, which can occur at multiple scales. While not explicitly identified as pericouplings in the literature, we found multiple examples of pericoupled systems in our literature review. For example, Risvoll et al. (2016) use interviews and participant observation to examine human–wildlife conflict between wild carnivores and pastoralists in the Nordland, Norway and identify challenges for cross-boundary management of wildlife between Sweden and Norway. This represents a pericoupling whereby wildlife (the flow) move between Norway and Sweden (receiving and sending systems).

The pericoupled movement of wildlife populations across geopolitical boundaries also occurs in marine systems, and may be increasing in frequency as climate change alters the distributions of commercially important species (Pinsky et al. 2018). For instance, Pacific cod (*Gadus microcephalus*), an important US export, are harvested from neighboring regions of Alaska and Russia and are likely genetically similar (Spies et al. 2020). However, they are currently managed independently by each national government. The mismatch between a pericoupled flow of fish between two systems, and independent governance of those two systems poses a threat to the sustainability of the fish populations in the long term.

In several instances of pericoupled animal migration, there have been bilateral or multilateral policies put in place to coordinate management, promote collaborative research, and ensure sustainable harvest of shared fisheries and wildlife populations. For example, the Chukchi Sea polar bear (*Ursus maritimus*) sub-population is co-managed by Indigenous communities and federal government representatives from both the USA and Russia who meet regularly to share knowledge and update policies to ensure the sustainability of this polar bear sub-population (U.S. Fish & Wildlife Service 2017). This sharing of information in and of itself represents a pericoupled information-sharing flow that is used to manage the pericoupled polar bear sub-population. Pericoupled (and telecoupled) information flows between the Arctic Range States are extremely common. Arctic countries have historically maintained a record of peaceful collaboration through intergovernmental forums and other organizations, such as the Arctic Council, the regional Barents Council, the Inuit Circumpolar Council, and others (Young 2016).

Pericoupled flows of humans and resources throughout the Arctic also allow for access to resources in isolated communities. At a sub-national scale, rural–urban transportation networks allow remote communities access to health care and other resources that are not available at home. Permanent or semi-permanent migration from rural to more urban or hub communities has also arisen as a concern in the Arctic and may exacerbate capacity building challenges in remote communities (Larsen and Fondahl 2015). Problems such as rising fuel costs and a lack of job opportunities have been cited as reasons for this phenomenon (Berman 2017). In particular, the loss of adult women and children from remote communities can result in the loss of key community assets, such as school buildings and the jobs and community gathering spaces they provide (Martin 2009).

### Arctic telecouplings

Telecoupling processes occur when human–nature interactions are separated across large distances (i.e., distant-coupled systems). While the Arctic is often thought of as a region isolated from the rest of the world, climatological, ecological, and social processes have long connected this region to lower latitudes. Multiple studies in our literature review examined “exogenous drivers” that would be classified as telecouplings when analyzed under the metacoupling framework (e.g., Meek 2011; Fauchald et al. 2017).

One prominent example of an Arctic telecoupling process occurring at an international scale is the concept of climatological teleconnections. In recent years, the Polar Vortex phenomenon, whereby Arctic air is transported south, has brought extreme cold spells and heavy precipitation to lower latitudes (Overland et al. 2016), and has gained much attention among both academic and non-academic audiences. While academics have primarily focused on the Polar Vortex phenomenon as a climatological event, it also has economic and social consequences in both the Arctic and lower latitudes. Examples of its socioeconomic effects in the northeastern USA include infrastructure damage, lengthy travel and transportation delays, and the loss of human life. When both the environmental phenomenon and its socioeconomic consequences are considered, the Polar Vortex can be viewed as a telecoupling process.

Beyond climate-related telecouplings, other telecoupling processes also connect the Arctic to lower latitudes. Long-distance species migrations, such as that of the Arctic tern (*Sterna paradisaea*), which can migrate up to 80 000 km between the Northern and Southern Hemispheres over the course of a year (Egevang et al. 2010), also connect Arctic and non-Arctic systems, with conservation and management implications throughout their migratory range.

Many telecoupling processes in the Arctic are ultimately connected to global markets. Global commodity prices are key drivers of natural resource development, including mining and oil and gas industries in Arctic countries (Arbo et al. 2013). This is particularly true for natural resource development projects in the Russian Arctic. Such processes include oil and gas drilling, commercial fishing, and mining. Gautier et al. (2009) estimate that up to 13% of the world’s undiscovered oil and 30% of its undiscovered gas may be located on or above the Arctic Circle. Additionally, walleye pollock (*Theragra chalcogramma*) harvested in the North Pacific make up 5% of total global fisheries and approximately 40% of US fisheries (Bailey et al. 1999). Despite the apparent outsized influence of global markets on Arctic natural resource development and their subsequent impacts on local communities, these connections appear to be relatively understudied in the Arctic CHANS literature. A notable exception, however, is Forbes (2013) who used intensive participant observation with nomadic Nenets reindeer herders in the Russian Arctic to examine the factors that influenced their resilience to climate change as well as land encroachment by large-scale oil and gas development. Forbes found that herder’s agency over their relatively small, privately held herds as well as flexible institutional oversight allowed for increased and rapid adaptability to the changing conditions and migration routes.

Tourism is another prominent telecoupling process occurring in many Arctic regions. In general, the phenomenon of “last chance tourism” has inspired many people to visit sites affected by climate change to see them “before they’re gone for good” (Lemelin et al. 2010). Visitors often travel to the Arctic to view iconic sights, such as glaciers, polar bears (*Ursus maritimus*), and the lights of the Aurora Borealis. While their travel brings money to Arctic nations, those funds often remain in the hands of large companies and infrequently benefit members of the communities that host the tourists (Maher et al. 2014), similar to the uneven distribution of benefits from tourism in remote nature reserves of lower latitudes (He et al. 2008). In addition to the carbon emissions associated with Arctic tourism (Dawson et al. 2010), there are also challenges associated with maintaining local biodiversity in heavily trafficked regions, as well as conflicts between land use for natural resource development and land use for tourism, such as the conflict between hydropower and tourism in Iceland (Saeþórsdóttir and Saarinen 2016).

## Interactions between metacouplings

Perhaps more important than any individual metacoupling process is the interaction between multiple, co-occurring metacoupling processes. The metacouplings discussed above (intracouplings, pericouplings, and telecouplings) rarely exist in isolation and are often interconnected with each other. These interconnections must be understood to accurately predict the cascading effects of any policy decision on Arctic sustainability. For example, the Covid-19 pandemic has spread throughout the world via telecoupled and pericoupled flows of travelers. However, in addition to the spread of the virus itself, the pandemic has had cascading consequences for telecoupled global supply chains (March et al. 2021), metacoupled economies (Pak et al. 2020), and intracoupled human–wildlife interactions (Shilling et al. 2021).

In many Arctic regions, a legacy effect of the extensive harmful impacts of previous pandemics led communities to take rapid actions that resulted in a delayed onset of the Covid-19 pandemic in many Arctic regions (Petrov et al. 2021). In this instance, historical experience with telecoupled transmission of diseases via flows of travelers was preserved through generations via intracoupled knowledge transfer and helped to mitigate some of the most devastating impacts of the current pandemic. In spite of these efforts, the Covid-19 pandemic has still had substantial cascading economic and social impacts on Arctic communities. Furthermore, scientific data collection in the Arctic, which is often conducted by scientists traveling from distant locations, has been hampered by the pandemic, resulting in a loss of critical observations and disruptions to data time series. However, this gap in research has also opened up opportunities and space for reflection on the benefits and importance of co-production of knowledge and long-standing, equitable partnerships between researchers and Arctic communities (Petrov et al. 2020).

While many studies in our review described different types of metacouplings, the lack of a consistent framework for examining these processes made it difficult to make generalizations or find patterns, leading to many broad-sweeping analyses of a diverse array of processes being non-differentially classified as “exogenous”. These different connections can result in various unintended consequences or emergent properties in CHANS. Thus, it is important to differentiate and interrelate them in future Arctic investigations.

For example, the economy of the Arctic is predominantly based on intracoupled natural resource extraction processes driven by telecoupled demand. Commercial fisheries, rare earth minerals (e.g., palladium), and hydrocarbons are all present in the Arctic, and increasing global demand has, in some cases, made them economically favorable for extraction (Glomsrød and Aslaksen 2006). These extractive industries, if unregulated, can pose a threat to the sustainability of Arctic ecosystems and the human communities with which they are interdependent.

In addition to incentivizing the flow of natural resources from the Arctic, telecoupling processes also play a role in local economies and local human–environment intracouplings by creating jobs that facilitate participation in the cash economy. For example, many rural communities rely on a mixed cash and subsistence economy (Kruse et al. 2008). A cash-based income allows for the purchase of materials and equipment needed for harvesting animals and plants. In Alaska, harvests are primarily used for household and community-level subsistence and traditional cultural practices of Indigenous communities. However, in other countries, such as Greenland (Denmark), wildlife harvest is primarily conducted by commercial hunters for distribution in local markets (Kruse et al. 2008). While it was originally hypothesized that Arctic residents would transition to an entirely cash economy with the introduction of market-driven jobs, the mixed economy has shown to be persistent and is predicted to remain in place (Burnsilver et al. 2016).

While previous research supports the idea that mixed economies are relatively stable (as opposed to a transitional state), the sustainability of rural communities has been drawn into question with regard to the phenomenon of rural–urban migration. This movement of individuals from rural to neighboring urban areas has been attributed to both the ability to participate in local, intracoupled subsistence activities (Berman 2009) and the presence of job opportunities in urban areas (Huskey et al. 2004). For example, using data from the survey of living conditions in the Arctic, Berman (2009) found that a decrease of 1% in harvest was significantly associated with a 1.25% increase in the probability of respondents considering moving away from a community. This finding is particularly relevant in the context of recent declines in Chinook salmon (*Oncorhynchus tshawytscha*) migrations along the Yukon River and subsequent impacts on community food security and wellbeing reported by media outlets (Hughes 2021). In addition to the lack of subsistence opportunities, in a review of rural–urban migration in Alaska Native communities, Huskey et al. (2004) found that economic opportunity was a key driver of migration. Understanding the relative influence of the intracoupled *push* of the lack of subsistence opportunities on the pericoupled migration process (i.e., sending system drives the pericoupled migration) versus the *pull* of job opportunities (i.e., receiving system drives the pericoupling) would provide critical knowledge needed to promote human wellbeing and sustainable livelihoods in the Arctic.

In the case of certain natural resources, such as fisheries, there is also the potential for conflict between intracoupling and telecoupling processes. Such is the case with commercial and subsistence fisheries in many parts of the Arctic. While subsistence fisheries make up a very small proportion of total harvest compared to commercial harvests (ca. 1% in Alaska), their cultural and economic importance has supported the creation of policies to ensure the ongoing ability of Indigenous communities to practice subsistence (Fall 2016). Subsistence-harvested species, although frequently shared among households or communities in a region, rarely leave the region in which they were harvested (Burnsilver et al. 2016). In the case of commercial fishing, the extraction of fish is driven by telecoupled demand and the fish, once captured, are transported through to distant, telecoupled markets. These distant connections lead to a complex web of interconnected costs and benefits that must be negotiated if sustainable and equitable solutions are to be found. Furthermore, while resource harvest for local use and consumption remains relatively small, the magnitude of processes linked to global trade, such as the walleye pollock fishery, is substantially larger.

Security and militarization in the Arctic provide another example of the interaction of multiple metacoupling processes. Relative to other global regions, the Arctic states have prided themselves on maintaining relatively peaceful relations and developing and promoting shared policy agendas through international forums such as the Arctic Council (Kankaanpää and Young 2012). This combination of pericoupled and telecoupled information sharing and policy development has led to important joint analyses and policies, such as the development of the Arctic Marine Shipping Assessment, which in turn influenced the development of the International Maritime Organization’s Polar Code (Arctic Council 2009; International Maritime Organization 2014) and Arctic state treaties on search and rescue, and oil spill preparedness and response.. Alongside these successes, however, have come increasing security challenges, particularly in light of increased economic development in the Russian maritime Arctic and the influence of declining sea ice extent on the opportunity for increased telecoupled marine transportation of Russian oil and gas to Asian and European markets (Brigham 2021). As has been the case in other regions, international trade can present challenges to the sustainability of CHANS in sending, receiving, and spillover systems (da Silva et al. 2017; Liu et al. 2018; Sun et al. 2018).

Telecoupled animal migration patterns can also have an influence on other metacouplings. For example, the short-tailed albatross (*Phoebastria albatrus*) only breeds on two islands in Japan but feeds in the North Pacific. Incidental catch of short-tailed albatrosses by commercial longline fisheries, such as the Pacific cod (*Gadus macrocephalus*) fishery in the Bering Sea, is the main source of human-caused mortalities in these birds. At a population size of ~1700 (BirdLife International 2018), human-caused mortalities have significant effects on its persistence. These incidental catches also have significant policy-driven implications for commercial fisheries. In the US’ North Pacific fisheries management system, an incidental catch of just 3 short-tailed albatrosses within a given year has the potential to shut down the entire longline fleet for the rest of that year (U.S. Fish & Wildlife Service 2008; U.S. Fish and Wildlife Service 2015). This is a case where a telecoupling process exists between the albatross’ breeding grounds (Japan) and feeding grounds (Bering Sea) through migration and international management, and the intracoupled action of exceeding permissible incidental catch (within the Bering Sea longline fleet) can have cascading consequences across other telecoupled systems (fisheries and the global market).

### Transportation of metacoupled flows

Previous research on telecoupling and metacoupling processes has often overlooked the social–ecological impacts of the transportation of metacoupled flows (Kapsar et al. 2019). Internationally traded commodities, natural resources, and other such physical flows are most commonly transported on large cargo or tanker ships. In fact, more than 80% of the world’s trade is transported in ships (UNCTAD 2017). In the Arctic, the majority of shipping is destinational (as opposed to trans-Arctic) and driven by global commodities prices (Arctic Council 2009). Ships carry resources from remote mining or oil and gas developments in the Arctic to distant refineries and processing facilities. Small and large cargo vessels are also used to transport supplies to remote coastal communities during summer sealift operations throughout all regions of the coastal Arctic Ocean.

In a metacoupling context, Arctic marine operations and shipping represent the primary ways in which the telecoupled flows of resources are transported into and out of the Arctic. When transiting between sending and receiving systems, these ships can have spillover effects on other systems through which they travel. These effects include noise pollution, the introduction of invasive species through ballast water contamination, the death of large-bodied cetaceans through ship strikes, and the interruption of subsistence practices (Robards et al. 2016).

The environmental, economic, and social impacts caused by the transportation of metacoupled flows contribute to the total impacts of metacoupled processes, such as international trade. Recent advances in ship tracking technology through the International Maritime Organization’s mandate of Automatic Identification System tracking technology on large vessels (> 300 gross tons), has facilitated an increased understanding of the spatial and temporal distribution of ships over the course of the last two decades (International Maritime Organization 2000). Researchers are increasingly applying Automatic Identification System data to map the distribution of the effects of shipping (Eguíluz et al. 2016; Meyers et al. 2021).

Metacoupled shipping processes in the Arctic have also precipitated the development of international policy frameworks for mitigating potential negative impacts. Entering into full force in 2018, the International Maritime Organization’s International Code for Ships Operating in Polar Waters was designed to provide a framework for enhancing marine safety and environmental protection. Also known as the Polar Code, these rules take a risk-based approach to enhance the safety of ship operations and prevent damage to both humans and the sensitive natural environment in polar waters (Deggim 2018). In a metacoupling context, the Polar Code seeks to minimize the spillover effects caused by the transportation of telecoupled flows of natural resources out of the Arctic.

### Climate change and the metacoupled Arctic

Climate change is arguably the single most pervasive force affecting metacoupling processes in the Arctic. This importance is reflected in the high prevalence of climate change studies in our literature review. With the Arctic warming at more than twice the global average rate (Overland et al. 2019), there are virtually no human or natural systems left unaffected. A changing Arctic climate not only alters local food webs and human–nature interactions (i.e., intracouplings; Moerlein and Carothers 2012; Cochran et al. 2013), but also has cascading impacts on global geopolitics. In particular, melting sea ice has increased marine access and created conditions whereby previously inaccessible natural resources are now economically favored for extraction. With the retreat of sea ice, shipping routes that could be used to transfer these resources to global markets are now opening (Smith and Stephenson 2013; Stephenson et al. 2013). These changes could result in the development of new telecoupling processes or the strengthening of existing ones.

In a more ecological context, research and concerns for the Arctic fox (*Vulpes lagopus*) provide a robust example of the effects of climate change on multiple metacoupling processes in the Arctic. As climate changes, boreal forests are expanding and the Arctic fox’s preferred habitat, tundra, is contracting (Selås et al. 2010). Climate change has also allowed for the northward expansion of the Arctic fox’s competitor, the red fox (*Vulpes vulpes*), causing a reduction in the Arctic fox’s range. As competitors, one would assume that these are part of normal ecological processes (e.g., competitive exclusion, predator–prey dynamics) affected by climate change. However, a study in Norway revealed that the red fox’s expansion is not only due to climate change, but also due to facilitation from an increase of human infrastructure (e.g., roads, cabins) for Arctic tourism, which increased food availability (e.g., human garbage; Selås et al. 2010). The Arctic fox now avoids human-occupied areas, not because of humans, but because of the red fox being present in those areas. Because of such patterns and effects, the Biodiversity Working Group of the Arctic Council has prioritized monitoring and the collection of information on this species (Berteaux et al. 2017). As a result, a total of 34 monitoring projects in Iceland, Greenland, Canada, the USA, Russia, Norway, Finland, and Sweden comprise a circumpolar monitoring system for this species. In sum, the effect of climate change in the Arctic has allowed for the expansion of red fox habitat (boreal forest) to an adjacent system (pericoupling), which aided the red fox’s expansion into this expanded habitat and was facilitated with the increase of tourism in the Arctic (telecouplings), causing competition within the system (intracoupling), and concerns for management of the Arctic fox in light of its range contraction has caused flows of information to be shared between and among adjacent and distant Arctic countries for international conservation efforts (peri- and telecouplings). Altogether, these form a complex web of metacoupling processes.

### Challenges for Arctic metacoupling research

When analyzing metacoupling processes, it is critical to define relevant boundaries and scale(s) of analysis. While defining scale is a fundamental challenge in both social and ecological systems research, it is generally recommended that to avoid scale mismatches, the scale of analysis should be proportional to the scale of the phenomena being studied (Cash et al. 2006; Cumming et al. 2012).

Defining boundaries can also be a challenge in metacoupled systems analysis. Boundaries can be defined based on ecological boundaries (e.g., permafrost, tree line, currents, species’ range), cultural or historical criteria (e.g., geographies of Indigenous lands or during a particular time period), or based on current political jurisdictions (e.g., among the eight Arctic **s**tates). The discussion of boundary definition is ongoing in the CHANS literature (Friis and Nielsen 2017; Liu et al. 2019). It is important to recognize that the choice of boundary can have substantial impacts upon the results of the analysis and that the decision of “membership” within a particular system may not be geographical (Friis and Nielsen 2017). For example, qualitative methods such as participant observation and interviews can be used to define membership within a given system from a network perspective. It is therefore critical to be clear as to the criteria used to define boundaries as well as the rationale behind the boundary definition.

Operationalizing the metacoupling framework for a particular system or flow involves the investment of resources to identify relevant components and metrics by which to define and measure them. As with all models, trade-offs exist between the level of effort or detail that is put into understanding a system and the degree of generalization of the model output. One can imagine that following every single flow of resources, material, or energy into, out of, or through a system would eventually result in a global-scale model so detailed that it would be rendered useless for any other purpose. In the case of metacoupled system models, we suggest that model elements are not useful if they do not impact the output of interest in a meaningful way. Techniques such as fuzzy cognitive mapping or system dynamics modeling can be used to determine relevant system components (Hobbs et al. 2002). It would be difficult to ensure that every relevant metacoupling component is identified in a given analysis. However, quantitative techniques (e.g., assessing model fit) or qualitative techniques (e.g., evaluating data saturation; Guest et al. 2020) may be used to ensure that a given model is comprehensive for its purpose. We point readers to recent overviews of modeling techniques and approaches in complex social–ecological systems for further discussion on this subject (Schlüter et al. 2019).

### Value of the metacoupling framework to Arctic sustainability research and policy

The metacoupling framework is an integrative tool that can be applied to better understand interactions within and among adjacent and distant CHANS. The qualitative skew in our review of Arctic CHANS literature reveals an opportunity for greater knowledge integration with quantitative, disciplinary evaluations of, for example, Arctic climate and ecology. Findings from specialized analyses or specific local knowledge can be better applied to decision-making when they are integrated and contextualized as part of a metacoupled system (Fidel et al. 2014). This integrated understanding can then be applied to better predict the ways in which a perturbation in one part of a system could have cascading effects on human–environment relationships in local, adjacent, and distant locations.

In individual studies, the application of the metacoupling framework can seem like a needless application of jargon. However, defining and labeling the different components of a metacoupled system fosters comparisons between different circumstances and disciplinary lenses. For example, in a comparative analysis of oil drilling impacts on Indigenous communities in Ecuador and Alaska, Haley (2004) demonstrated that the tight-knit and cohesive community of agents in Arctic Alaska was a critical element that allowed for Alaska Native communities on the North Slope to advocate for the consideration of subsistence practices in planning for natural resource development for global trade (Haley 2004). However, the lack of cohesion among Indigenous agents in Ecuador led to less successful negotiation practices. In this case, the social cohesion among the agents in Arctic Alaska was a cause that resulted in a modification of the flow of natural resources from the North Slope during subsistence seasons, thus modifying a telecoupling process (natural resource extraction for global trade) in order to maintain an existing intracoupling process (subsistence). When placed in the framework of metacoupling, these findings present a generalizable hypothesis that the facilitation of social cohesion and capacity building among local actors navigating telecoupling processes is a critical element to ensure that local concerns are addressed, and mutually beneficial solutions are developed. This hypothesis could then be tested in other settings or expanded to determine whether the same principles hold true, for example, to ask whether social cohesion among actors is critical for the sustainable maintenance of intracoupling processes in general.

The metacoupling framework also allows for more systematic analyses of CHANS through the identification of knowledge gaps with regard to their constituent components and the relationships between them. Table 2 gives an example of an application of the metacoupling framework to Parlee et al.’s analysis of the threats of mining to the Bathurst barren ground caribou herd (*Rangifer tarandus groenlandicus*; Parlee et al. 2018). This application shows that while there is a body of traditional and scientific knowledge demonstrating the impacts of mining activities on caribou herds and subsistence practices, there has been less CHANS research examining the drivers of mining or the motivations and decision-making structures of certain actors, such as mining corporations. Further research is needed to determine whether these gaps are covered in other studies (and thus a source for future knowledge syntheses) or are areas for future research.

**Table 2:** Application of the metacoupling framework to identify future research directions on the effects of mining on Bathurst barren ground caribou (*Rangifer tarandus groenlandicus*) herd, based on a reading of Parlee et al. 2018 as an example. Underlined items indicate metacoupling components not analyzed within the scope of the study

Components of the metacoupling framework	Specific metacoupling component analyzed or not analyzed (Parlee et al. 2018—Bathurst Caribou Herd)
Sending systems	Range of the Bathurst caribou herd (including Ekati and Diavik Mines and the “Jay Project”)
Telecoupled receiving systems	Systems where minerals are utilized
Pericoupled receiving systems	Systems through which minerals are transported and/or processed
Spillover systems	Systems involved in mineral refinement; Systems of origin for non-Indigenous hunters using mining roads to harvest caribou
Flows	Migration of caribou from calving grounds in Bathurst Inlet to central Northwest Territories; Policies banning hunting for Indigenous hunters; Movement of minerals
Agents	Dene First Nation communities; Mining corporations; Governments (e.g., Northwest Territories Environment and Resources)
Causes	Government approval of extraction; Land ownership practices; Lack of communication, trust, and power-sharing between governments and Indigenous communities; demand for mineral resources
Effects	Loss of caribou habitat; Decline of caribou population; Inability to practice subsistence; Increased use of alternative food sources; Altered caribou migration patterns

Additionally, while this paper focuses on environmental and economic aspects of Arctic CHANS, the metacoupling framework could be used to examine other areas of research, such as education. For example, the metacoupling framework could be used to evaluate the relative influence of culturally sensitive approaches and education in traditional knowledge in contrast with western education, and their increasing mix in local communities on the wellbeing of Arctic residents and ecological systems.

The application of the metacoupling framework to Arctic CHANS can not only improve research, but also assist with the development of more effective Arctic sustainability policies. Identifying the various systems, agents, flows, causes, and effects that situate Arctic CHANS within complex metacoupled systems increases transparency, which is a first step toward effective policymaking (Munroe et al. 2019). Additionally, understanding the Arctic as a metacoupled system can also help in the development of polycentric governance processes that coordinate governance in the Arctic with adjacent and distant systems (Oberlack et al. 2018). For example, identifying the ecosystem services provided by migratory species, such as the pest control services provided by Mexican free-tailed bats (*Tadarida brasiliensis*) in the USA (López-hoffman et al. 2017), could assist with ensuring equity in the transboundary management of migratory wildlife. Transboundary management is particularly important for the Arctic where many species of subsistence importance migrate from distant locales where they face substantial anthropogenic threats that in turn affect population dynamics in their Arctic summering grounds.

## Conclusion

Much interdisciplinary CHANS research has been conducted to better understand human–nature interactions in the Arctic. Similarly, many disciplinary studies have analyzed the relationship between the Arctic and lower latitudes. However, these studies are often conducted in isolation and at relatively small scales. Holistic approaches to understanding complex systems could assist with knowledge integration to better place Arctic sustainability in a global context. This review highlights the utility of the metacoupling framework for integrating knowledge across scales and from multiple areas of study into a more complete understanding of Arctic CHANS in a globalized world. The metacoupling framework could be used to guide researchers in identifying knowledge gaps in their study system or areas for knowledge synthesis by answering questions such as: what socioeconomic or environmental flows connect a focal system to distant systems? Who are the actors involved in perpetuating or weakening these flows? How might actions in distant systems affect the sustainability of a certain aspect of the focal system? How do actors or flows in the focal study system impact other systems that are nearby or far away. Answers to these questions can be used to facilitate knowledge integration and to create comprehensive and effective policies for promoting sustainability objectives while minimizing negative unintended consequences.

## Supplementary Information

Below is the link to the electronic supplementary material.

Supplementary file1 (PDF 188 kb)
