# Supplementary material for: The metacoupled Arctic: Human–nature interactions across local to global scales as drivers of sustainability
Source: Ambio. 2022 Mar 30;51(10):2061–78. doi: 10.1007/s13280-022-01729-9 (PMC9378800; doi:10.1007/s13280-022-01729-9)
Supplement: Supplementary file 1 — Supplementary file1 (PDF 188 kb) [file 13280_2022_1729_MOESM1_ESM.pdf]

AMBIO

Electronic Supplementary Material
This supplementary material has not been peer reviewed.

Title: **The metacoupled Arctic: Human-nature interactions across local to global scales as drivers of sustainability**

Table S1 Variable names and definitions used in the literature review of Arctic CHANS analyses. These fields correspond with the data provided in Table S3.

**Paper Data Category**	**Description**
Number of Countries Studied	Indicate whether one or more countries were studied. Also includes specifications for entirely high seas or marine analyses that do not consider country boundaries.
Country/Countries Studied	Countries from which data or other information were collected for the purposes of the analysis conducted in the article.
Type of Research	Indicate whether the data or other information that was collected for the purposes of the analysis conducted in the article were qualitative, quantitative, or a combination of the two.
Single scale or multi-scale research	Determine whether the collected data were analyzed at multiple geographic scales (e.g., at community and regional scales) or aggregated into a single scale of analysis.
Geographic extent of research	The largest scale that encompasses the geographic range at which data were collected. For example, a study involving a survey of three communities within a country would be considered regional, while an analysis of three communities in two different countries would be considered international.
Community involvement	Indicate whether local communities were involved in the research process. We distinguished between involvement as participants (e.g., in a survey/focus group) and involvement in the design and/or approach of the research (e.g., participatory methods).
External influences	Indicate whether an external or exogenous influence was a primary focus of the analysis of the study system. The definition of "external" was relative to the description and boundaries of the study area as described in the paper.

Table S2 Studies included in the systematic literature review of Arctic coupled human and natural systems analyses.

Alessa, L. (Na ia), A. (Anaru) Kliskey, R. Busey, L. Hinzman, and D. White. 2008. Freshwater vulnerabilities and resilience on the Seward Peninsula: Integrating multiple dimensions of landscape change. *Global Environmental Change* 18: 256–270. doi:10.1016/j.gloenvcha.2008.01.004.

Alessa, L. (Na ia), A. (Anaru) Kliskey, P. Williams, and M. Barton. 2008. Perception of change in freshwater in remote resource-dependent Arctic communities. *Global Environmental Change* 18: 153–164. doi:10.1016/j.gloenvcha.2007.05.007.

Alessa, L. (Naia), A. (Anaru) Kliskey, and G. Brown. 2008. Social-ecological hotspots mapping: A spatial approach for identifying coupled social-ecological space. *Landscape and Urban Planning* 85: 27–39. doi:10.1016/j.landurbplan.2007.09.007.

Alessa, L. (Naia), A. (Anaru) Kliskey, and P. Williams. 2010. Forgetting freshwater: Technology, values, and distancing in remote arctic communities. *Society and Natural Resources* 23: 254–268. doi:10.1080/08941920802454813.

Alessa, L., A. Kliskey, R. Lammers, C. Arp, D. White, L. Hinzman, and R. Busey. 2008. The arctic water resource vulnerability index: An integrated assessment tool for community resilience and vulnerability with respect to freshwater. *Environmental Management* 42: 523–541. doi:10.1007/s00267-008-9152-0.

Altaweel, M. R., L. N. Alessa, and A. D. Kliskey. 2009. Forecasting Resilience in arctic societies: Creating tools for assessing social-hydrological systems. *Journal of the American Water Resources Association* 45: 1379–1389. doi:10.1111/j.1752-1688.2009.00370.x.

Amundsen, H. 2012. Illusions of resilience? An analysis of community responses to change in northern Norway. *Ecology and Society* 17. doi:10.5751/ES-05142-170446.

Andrachuk, M., and B. Smit. 2012. Community-based vulnerability assessment of Tuktoyaktuk, NWT, Canada to environmental and socio-economic changes. *Regional Environmental Change* 12: 867–885. doi:10.1007/s10113-012-0299-0.

Armitage, D. R. 2005. Community-based Narwhal management in Nunavut, Canada: Change, uncertainty, and adaptation. *Society and Natural Resources* 18: 715–731. doi:10.1080/08941920591005124.

Armitage, D., F. Berkes, A. Dale, E. Kocho-Schellenberg, and E. Patton. 2011. Co-management and the co-production of knowledge: Learning to adapt in Canada's Arctic. *Global Environmental Change* 21. Elsevier Ltd: 995–1004. doi:10.1016/j.gloenvcha.2011.04.006.

Baggio, J. A., S. B. Burnsilver, A. Arenas, J. S. Magdanz, G. P. Kofinas, and M. De Domenico. 2016. Multiplex social ecological network analysis reveals how social changes affect community robustness more than resource depletion. *Proceedings of the National Academy of Sciences of the United States of America* 113: 13708–13713. doi:10.1073/pnas.1604401113.

Bali, A., and G. P. Kofinas. 2014. Voices of the Caribou people: A participatory videography method to document and share local knowledge from the North American human- Rangifer systems. *Ecology and Society* 19. doi:10.5751/ES-06327-190216.

Bay-Larsen, I., C. Risvoll, I. Vestrum, and H. Bjørkhaug. 2018. Local protein sources in animal feed - Perceptions among arctic sheep farmers. *Journal of Rural Studies* 59: 98–110. doi:10.1016/j.jrurstud.2018.02.004.

Berkes, F., and D. Jolly. 2002. Adapting to climate change: social-ecological resilience in a Canadian western Arctic community. *Conservation ecology* 5: 18.
Blair, B., and A. L. Lovecraft. 2020. Risks without borders: A cultural consensus model of risks to sustainability in rapidly changing social-ecological systems. *Sustainability (Switzerland)* 12. doi:10.3390/su12062446.
Brinkman, T., K. B. Maracle, J. Kelly, M. Vandyke, A. Firmin, and A. Springsteen. 2014. Impact of fuel costs on high-latitude subsistence activities. *Ecology and Society* 19: 18. doi:10.5751/ES-06861-190418.
Broderstad, E. G., and E. Eythórsson. 2014. Resilient communities? Collapse and recovery of a social-ecological system in Arctic Norway. *Ecology & Society* 19: 1.
Burgass, M. J., E. J. Milner-Gulland, J. S. Stewart Lowndes, C. O'Hara, J. C. Afflerbach, and B. S. Halpern. 2018. A pan-Arctic assessment of the status of marine social-ecological systems. *Regional Environmental Change* 19: 293–308. doi:10.1007/s10113-018-1395-6.
Burkhard, B., and F. Müller. 2008. Indicating human-environmental system properties: Case study northern Fenno-Scandinavian reindeer herding. *Ecological Indicators* 8: 828–840. doi:10.1016/j.ecolind.2007.06.003.
Carothers, C. 2010. Tragedy of commodification: Displacements in Alutiiq Fishing Communities in the Gulf of Alaska. *Mast* 9: 95–120. doi:10.1017/CBO9781107415324.004.
Chapin, F. S., S. F. Trainor, O. Huntington, A. L. Lovecraft, E. Zavaleta, D. C. Natcher, A. D. McGuire, J. L. Nelson, et al. 2008. Increasing wildfire in Alaska's boreal forest: Pathways to potential solutions of a wicked problem. *BioScience* 58: 531–540. doi:10.1641/B580609.
Cline, T. J., D. E. Schindler, and R. Hilborn. 2017. Fisheries portfolio diversification and turnover buffer Alaskan fishing communities from abrupt resource and market changes. *Nature Communications* 8: 14042. Nature Publishing Group. doi:10.1038/ncomms14042.
Colavito, M. M., S. F. Trainor, N. P. Kettle, and A. York. 2019. Making the Transition from Science Delivery to Knowledge Coproduction in Boundary Spanning: A Case Study of the Alaska Fire Science Consortium. *Weather, Climate, and Society* 11: 917–934. doi:10.1175/wcas-d-19-0009.1.
Colloff, M. J., R. M. Wise, I. Palomo, S. Lavorel, and U. Pascual. 2020. Nature's contribution to adaptation: insights from examples of the transformation of social-ecological systems. *Ecosystems and People* 16: 137–150. doi:10.1080/26395916.2020.1754919.
Courault, R., and M. Cohen. 2020. Evolution of Land Cover and Ecosystem Services in the Frame of Pastoral Functional Categories: A Case Study in Swedish Lapland. *Sustainability* 12: 390. doi:10.3390/su12010390.
Crépin, A.-S., A. Gren, G. Engstrom, and D. Ospina. 2017. Operationalising a social – ecological system perspective on the Arctic Ocean. *Ambio* 46: 475-485. doi:10.1007/s13280-017-0960-4.
Dammann, D. O., H. Eicken, A. R. Mahoney, F. J. Meyer, and S. Betcher. 2018. Assessing sea ice trafficability in a changing arctic. *Arctic* 71: 59–75. doi:10.14430/arctic4701.
Dannevig, H., I. Bay-Larsen, B. van Oort, and E. C. H. Keskitalo. 2015. Adaptive capacity to changes in terrestrial ecosystem services amongst primary small-scale resource users in northern Norway and Sweden. *Polar Geography* 38: 271–288. doi:10.1080/1088937X.2015.1114533.
Debortoli, N. S., J. S. Sayles, D. G. Clark, and J. D. Ford. 2018. A systems network approach for climate change vulnerability assessment. *Environmental Research Letters* 13: 104019. doi:10.1088/1748-9326/aae24a.

Druckenmiller, M. L., H. Eicken, J. C. C. George, and L. Brower. 2013. Trails to the whale: Reflections of change and choice on an Iñupiat icescape at Barrow, Alaska. *Polar Geography* 36: 5–29. doi:10.1080/1088937X.2012.724459.

Ehrich, D., A. E. Thuestad, H. Tømmervik, P. Fauchald, and V. H. Hausner. 2019. Local land use associated with socio-economic development in six arctic regions. *Ambio* 48: 649–660. doi:10.1007/s13280-018-1095-y.

Falardeau, M., C. Raudsepp-Hearne, and E. M. Bennett. 2019. A novel approach for co-producing positive scenarios that explore agency: case study from the Canadian Arctic. *Sustainability Science* 14: 205–220. doi:10.1007/s11625-018-0620-z.

Fauchald, P., V. H. Hausner, J. I. Schmidt, and D. A. Clark. 2017. Transitions of social-ecological subsistence systems in the Arctic. *International Journal of the Commons* 11: 275–329. doi:10.18352/ijc.698.

Fedreheim, G. E., and E. Blanco. 2017. Co-management of protected areas to alleviate conservation conflicts: Experiences in Norway. *International Journal of the Commons* 11: 754–773. doi:10.18352/ijc.749.

Fidel, M., A. Kliskey, L. Alessa, and O. (Olia) P. Sutton. 2014. Walrus harvest locations reflect adaptation: a contribution from a community-based observation network in the Bering Sea. *Polar Geography* 37: 48–68. doi:10.1080/1088937X.2013.879613.

Flint, C. G., E. S. Robinson, J. Kellogg, G. Ferguson, L. BouFajreldin, M. Dolan, I. Raskin, and M. A. Lila. 2011. Promoting wellness in Alaskan villages: Integrating traditional knowledge and science of wild berries. *EcoHealth* 8: 199–209. doi:10.1007/s10393-011-0707-9.

Forbes, B. C., F. Stammler, T. Kumpula, N. Meschtyb, A. Pajunen, and E. Kaarlejarvi. 2009. High resilience in the Yamal-Nenets social-ecological system, West Siberian Arctic, Russia. *Proceedings of the National Academy of Sciences* 106: 22041–22048. doi:10.1073/pnas.0908286106.

Forbes, B. C. 2013. Cultural resilience of social-ecological systems in the Nenets and Yamal-Nenets Autonomous Okrugs, Russia: A focus on reindeer nomads of the tundra. *Ecology and Society* 18: 36. doi:10.5751/ES-05791-180436.

Forbes, B. C., T. Kumpula, N. Meschtyb, R. Laptander, M. MacIas-Fauria, P. Zetterberg, M. Verdonen, A. Skarin, et al. 2016. Sea ice, rain-on-snow and tundra reindeer nomadism in Arctic Russia. *Biology Letters* 12: 4–8. doi:10.1098/rsbl.2016.0466.

Forbes, B. C., M. T. Turunen, P. Soppela, S. Rasmus, T. Vuojala-Magga, and H. Kitti. 2020. Changes in mountain birch forests and reindeer management: Comparing different knowledge systems in Sápmi, northern Fennoscandia. *Polar Record* 55: 507-521. doi:10.1017/S0032247419000834.

Ford, J. D. 2009. Vulnerability of Inuit food systems to food insecurity as a consequence of climate change: A case study from Igloolik, Nunavut. *Regional Environmental Change* 9: 83–100. doi:10.1007/s10113-008-0060-x.

Ford, J. D., G. McDowell, J. Shirley, M. Pitre, R. Siewierski, W. Gough, F. Duerden, T. Pearce, et al. 2013. The Dynamic Multiscale Nature of Climate Change Vulnerability: An Inuit Harvesting Example. *Annals of the Association of American Geographers* 103: 1193–1211. doi:10.1080/00045608.2013.776880.

Galappaththi, E. K., J. D. Ford, E. M. Bennett, and F. Berkes. 2019. Climate change and community fisheries in the arctic: A case study from Pangnirtung, Canada. *Journal of Environmental Management* 250: 109534. doi:10.1016/j.jenvman.2019.109534.

Gill, H., T. Lantz, and the Gwich'in Social and Cultural Institute. 2014. A Community-Based Approach to Mapping Gwich'in Observations of Environmental Changes in the Lower Peel River Watershed , NT. *Journal of Ethnobiology* 34: 294–314. doi:http://dx.doi.org/10.2993/0278-0771-34.3.294.

Gondor, D. 2016. Inuit knowledge and environmental assessment in Nunavut, Canada. *Sustainability Science* 11: 153–162. doi:10.1007/s11625-015-0310-z.

Hamilton, L. C., S. Jónsson, H. Ögmundardóttir, and I. M. Belkin. 2004. Sea changes ashore: The ocean and iceland's herring capital. *Arctic* 57: 325–335. doi:10.14430/arctic511.

Hansen, W. D., T. J. Brinkman, F. S. Chapin, and C. Brown. 2013. Meeting Indigenous Subsistence Needs: The Case for Prey Switching in Rural Alaska. *Human Dimensions of Wildlife* 18: 109–123. doi:10.1080/10871209.2012.719172.

Hansen, W. D., T. J. Brinkman, M. Leonawicz, F. S. Chapin, and G. P. Kofinas. 2013. Changing Daily Wind Speeds on Alaska's North Slope: Implications for Rural Hunting Opportunities. *Arctic* 66: 448–458.

Hausner, V. H., P. Fauchald, and J. L. Jernsletten. 2012. Community-Based Management: Under What Conditions Do Sámi Pastoralists Manage Pastures Sustainably? *PLoS ONE* 7: e51187. doi:10.1371/journal.pone.0051187.

Hausner, V. H., P. Fauchald, T. Tveraa, E. Pedersen, J. L. Jernsletten, B. Ulvevadet, R. A. Ims, N. G. Yoccoz, et al. 2011. The ghost of development past: The impact policies on saami pastoral ecosystems. *Ecology and Society* 16: 07. doi:10.5751/ES-04193-160304.

Hillmer-Pegram, K. 2016. Integrating Indigenous values with capitalism through tourism: Alaskan experiences and outstanding issues. *Journal of Sustainable Tourism* 24: 1194–1210. doi:10.1080/09669582.2016.1182536.

Himes-Cornell, A., and S. Kasperski. 2015. Assessing climate change vulnerability in Alaska's fishing communities. *Fisheries Research* 162: 1–11. doi:10.1016/j.fishres.2014.09.010.

Himes-Cornell, A., C. Maguire, S. Kasperski, K. Hoelting, and R. Pollnac. 2016. Understanding vulnerability in Alaska fishing communities: A validation methodology for rapid assessment of indices related to well-being. *Ocean and Coastal Management* 124: 53–65. doi:10.1016/j.ocecoaman.2016.02.004.

Huntington, H. P., S. A. Kruse, and A. J. Scholz. 2009. Demographic and environmental conditions are uncoupled in the social-ecological system of the Pribilof Islands. *Polar Research* 28: 119–128. doi:10.1111/j.1751-8369.2009.00096.x.

Jansson, R., C. Nilsson, E. C. H. Keskitalo, T. Vlasova, M. L. Sutinen, J. Moen, F. Stuart Chapin, K. A. Bråthen, et al. 2015. Future changes in the supply of goods and services from natural ecosystems: Prospects for the European North. *Ecology and Society* 20: 3. doi:10.5751/ES-07607-200332.

Johansson, K., and M. Manseau. 2012. Inuit Safety Culture and Its Relevance to Safety Management in Auyuittuq National Park. *Society and Natural Resources* 25: 176–183. doi:10.1080/08941920.2010.551533.

Jones, C. E., K. Kielland, L. D. Hinzman, and W. S. Schneider. 2015. Integrating local knowledge and science: economic consequences of driftwood harvest in a changing climate. *Ecology and Society* 20: 1. doi:10.5751/ES-07235-200125.

Kassam, K. A. S. 2010. Coupled socio-cultural and ecological systems at the margins: Arctic and alpine cases. *Frontiers of Earth Science in China* 4: 89–98. doi:10.1007/s11707-010-0008-6.

Kettle, N. P. 2019. Knowledge co-production in contested spaces: An evaluation of the north slope borough-shell baseline studies program. *Arctic* 72: 43–57. doi:10.14430/arctic67804.
Klokov, K. B. 2013. Changes in reindeer population numbers in Russia: an effect of the political context or of climate? *Rangifer* 2: 19. doi:10.7557/2.32.1.2234.
Koenigstein, S., M. Ruth, and S. Gößling-Reisemann. 2016. Stakeholder-informed ecosystem modeling of ocean warming and acidification impacts in the barents sea region. *Frontiers in Marine Science* 3: 1–13. doi:10.3389/fmars.2016.00093.
Kokorsch, M., and K. Benediktsson. 2018. Prosper or perish? The development of Icelandic fishing villages after the privatisation of fishing rights. *Maritime Studies* 17: 69–83. doi:10.1007/s40152-018-0089-5.
Kokorsch, M., and K. Benediktsson. 2018. Where have all the people gone? The limits of resilience in coastal communities. *Norsk Geografisk Tidsskrift* 72: 97–114. doi:10.1080/00291951.2018.1450289.
Kryazhimskii, F. V., K. V. Maklakov, L. M. Morozova, and S. N. Ektova. 2011. System analysis of biogeocenoses of the Yamal Peninsula: Simulation of the impact of large-herd reindeer breeding on vegetation. *Russian Journal of Ecology* 42: 351–361. doi:10.1134/S1067413611050092.
Kumpula, T., B. C. Forbes, F. Stammler, and N. Meschtyb. 2012. Dynamics of a coupled system: Multi-resolution remote sensing in assessing social-ecological responses during 25 years of gas field development in Arctic Russia. *Remote Sensing* 4: 1046–1068. doi:10.3390/rs40401046.
Kumpula, T., A. Pajunen, E. Kaarlejärvi, B. C. Forbes, and F. Stammler. 2011. Land use and land cover change in Arctic Russia: Ecological and social implications of industrial development. *Global Environmental Change* 21: 550–562. doi:10.1016/j.gloenvcha.2010.12.010.
Lamalice, A., D. Haillot, M. A. Lamontagne, T. M. Herrmann, S. Gibout, S. Blangy, J. L. Martin, V. Coxam, et al. 2018. Building Food Security in the Canadian Arctic through the Development of Sustainable Community Greenhouses and Gardening. *Ecoscience* 25: 325–341. doi:10.1080/11956860.2018.1493260.
Loring, P. A. 2016. Toward a Theory of Coexistence in Shared Social-Ecological Systems: The Case of Cook Inlet Salmon Fisheries. *Human Ecology* 44: 153–165. doi:10.1007/s10745-016-9806-0.
Loring, P. A., S. C. Gerlach, and H. J. Penn. 2016. "Community work" in a climate of adaptation: Responding to change in rural Alaska. *Human Ecology* 44: 119–128. doi:10.1007/s10745-015-9800-y.
Lovvorn, J. R., A. R. Rocha, A. H. Mahoney, and S. C. Jewett. 2018. Sustaining ecological and subsistence functions in conservation areas: Eider habitat and access by Native hunters along landfast ice. *Environmental Conservation* 45: 361–369. doi:10.1017/S0376892918000103.
Lyons, C., C. Carothers, and K. Reedy. 2016. Means, meanings, and contexts: A framework for integrating detailed ethnographic data into assessments of fishing community vulnerability. *Marine Policy* 74: 341–350. doi:10.1016/j.marpol.2016.04.022.
Martin, S. 2015. Indigenous social and economic adaptations in northern Alaska as measures of resilience. *Ecology and Society* 20: 8. doi:10.5751/ES-07586-200408.

McDowell, G., and J. D. Ford. 2014. The socio-ecological dimensions of hydrocarbon development in the Disko Bay region of Greenland: Opportunities, risks, and tradeoffs. *Applied Geography* 46: 98–110. doi:10.1016/j.apgeog.2013.11.006.

McNeeley, S. M., and M. D. Shulski. 2011. Anatomy of a closing window: Vulnerability to changing seasonality in Interior Alaska. *Global Environmental Change* 21: 464–473. doi:10.1016/j.gloenvcha.2011.02.003.

Meek, C. L. 2011. Putting the US polar bear debate into context: the disconnect between old policy and new problems. *Marine Policy* 35: 430–439.

Meek, C. L. 2013. Forms of collaboration and social fit in wildlife management: A comparison of policy networks in Alaska. *Global Environmental Change* 23: 217–228.

Moerlein, K. J., and C. Carothers. 2012. Total Environment of Change: Impacts of Climate Change and Social Transitions on Subsistence Fisheries in Northwest Alaska. *Ecology and Society* 17: 1. doi:10.5751/es-04543-170110.

Nicolson, C., M. Berman, C. Thor West, G. P. Kofinas, B. Griffith, D. Russell, and D. Dugan. 2013. Seasonal climate variation and caribou availability: Modeling sequential movement using satellite-relocation data. *Ecology and Society* 18: 2. doi:10.5751/ES-05376-180201.

Parlee, B. L., J. Sandlos, and D. C. Natcher. 2018. Undermining subsistence: Barren-ground caribou in a "tragedy of open access." *Science Advances* 4: 1–15. doi:10.1126/sciadv.1701611.

Parlee, B., F. Berkes, and T. Gwich'In. 2005. Health of the land, health of the people: A case study on Gwich'in Berry harvesting in Northern Canada. *EcoHealth* 2: 127–137. doi:10.1007/s10393-005-3870-z.

Planque, B., C. Mullon, P. Arneberg, A. Eide, J. M. Fromentin, J. J. Heymans, A. H. Hoel, S. Niiranen, et al. 2019. A participatory scenario method to explore the future of marine social-ecological systems. *Fish and Fisheries* 20: 434–451. doi:10.1111/faf.12356.

Proverbs, T. A., T. C. Lantz, S. I. Lord, A. Amos, and N. C. Ban. 2020. Social-Ecological Determinants of Access to Fish and Well-Being in Four Gwich'in Communities in Canada's Northwest Territories. *Human Ecology* 48: 155–171. doi:10.1007/s10745-020-00131-x.

Rathwell K.J., A. D., and R. K.J. 2016. Art and artistic processes bridge knowledge systems about social-ecological change: An empirical examination with Inuit artists from Nunavut, Canada. *Ecology and Society* 21: 2. doi:10.5751/ES-08369-210221.

Rattenbury, K., K. Kielland, G. Finstad, and W. Schneider. 2009. A reindeer herder's perspective on caribou, weather and socio-economic change on the Seward Peninsula, Alaska. *Polar Research* 28: 71–88. doi:10.1111/j.1751-8369.2009.00102.x.

Risvoll, C., G. E. Fedreheim, and D. Galafassi. 2016. Trade-offs in pastoral governance in Norway: Challenges for biodiversity and adaptation. *Pastoralism* 6: 4. doi:10.1186/s13570-016-0051-3.

Risvoll, C., G. E. Fedreheim, A. Sandberg, and S. BurnSilver. 2014. Does Pastoralists' participation in the management of national parks in northern Norway contribute to adaptive governance? *Ecology and Society* 19: 2. doi:10.5751/ES-06658-190271.

Risvoll, C., and G. K. Hovelsrud. 2016. Pasture access and adaptive capacity in reindeer herding districts in Nordland, Northern Norway. *Polar Journal* 6: 87–111. doi:10.1080/2154896X.2016.1173796.

Rutherford, T. K., and C. A. Schultz. 2019. Adapting wildland fire governance to climate change in Alaska. *Ecology and Society* 24: 1. doi:10.5751/ES-10810-240127.

Rybråten, S., M. Bjørkan, G. K. Hovelsrud, and B. P. Kaltenborn. 2018. Sustainable coasts? Perceptions of change and livelihood vulnerability in nordland, norway. *Local Environment* 23: 1156–1171. doi:10.1080/13549839.2018.1533931.

Sarkki, S., K. Latola, M. Jokinen, and A. Stepien. 2014. Socio-Natural Capital for Sustainable Land Use in the Fennoscandia. *Arctic Yearbook*: 1–16.

Stamberger, L., C. J. van Riper, R. Keller, M. Brownlee, and J. Rose. 2018. A GPS tracking study of recreationists in an Alaskan protected area. *Applied Geography* 93: 92–102. doi:10.1016/j.apgeog.2018.02.011.

Takakura, H. 2016. Limits of pastoral adaptation to permafrost regions caused by climate change among the Sakha people in the middle basin of Lena River. *Polar Science* 10: 395–403. doi:10.1016/j.polar.2016.04.003.

Trainor, S. F., M. Calef, D. Natcher, F. S. Chapin, A. D. Mcguire, O. Huntington, P. Duffy, T. S. Rupp, et al. 2009. Vulnerability and adaptation to climate-related fire impacts in rural and urban interior Alaska. *Polar Research* 28: 100–118. doi:10.1111/j.1751-8369.2009.00101.x.

Tremblay, R., M. Landry-Cuerrier, and M. M. Humphries. 2020. Culture and the social-ecology of local food use by indigenous communities in northern North America. *Ecology and Society* 25: 1–26. doi:10.5751/ES-11542-250208.

Turi, E. I., and E. C. H. Keskitalo. 2014. Governing reindeer husbandry in western Finnmark: barriers for incorporating traditional knowledge in local-level policy implementation. *Polar Geography* 37: 234–251. doi:10.1080/1088937X.2014.953620.

Vihervaara, P., T. Kumpula, A. Tanskanen, and B. Burkhard. 2010. Ecosystem services-A tool for sustainable management of human-environment systems. Case study Finnish Forest Lapland. *Ecological Complexity* 7: 410–420. doi:10.1016/j.ecocom.2009.12.002.

West, C. T. 2011. The survey of living conditions in the Arctic (SLiCA): A comparative sustainable livelihoods assessment. *Environment, Development and Sustainability* 13: 217–235. doi:10.1007/s10668-010-9257-5.

West, J. J., and G. K. Hovelsrud. 2010. Cross-scale adaptation challenges in the coastal fisheries: Findings from Lebesby, Northern Norway. *Arctic* 63: 338–354. doi:10.14430/arctic1497.

Wheeler, H. C., D. Berteaux, C. Furgal, K. Cazelles, N. G. Yoccoz, and D. Grémillet. 2019. Identifying key needs for the integration of social–ecological outcomes in arctic wildlife monitoring. *Conservation Biology* 33: 861-872. doi:10.1111/cobi.13257.

Williams, P., A. Kliskey, M. McCarthy, R. Lammers, L. Alessa, and J. Abatzoglou. 2019. Using the Arctic water resources vulnerability index in assessing and responding to environmental change in Alaskan communities. *Climate Risk Management* 23: 19–31. doi:10.1016/j.crm.2018.09.001.

Wilson, N. J. 2014. The Politics of Adaptation: Subsistence Livelihoods and Vulnerability to Climate Change in the Koyukon Athabascan Village of Ruby, Alaska. *Human Ecology* 42: 87–101. doi:10.1007/s10745-013-9619-3.

Wilson, N. J., M. T. Walter, and J. Waterhouse. 2017. Indigenous Knowledge of Hydrologic Change in the Yukon River Basin: A Case Study. *Arctic* 68: 93–106.

Young, R. C., A. S. Kitaysky, C. Carothers, and I. Dorresteijn. 2014. Seabirds as a subsistence and cultural resource in two remote Alaskan communities. *Ecology and Society* 19: 4. doi:10.5751/ES-07158-190440.
